# Effectiveness of Nationwide Screening Program for Neuroblastoma in Japan

**DOI:** 10.5539/gjhs.v6n4p94

**Published:** 2014-04-10

**Authors:** Akinori Hisashige, for the Group NBS Evaluation

**Affiliations:** 1 The Institute of Healthcare Technology Assessment, Tokushima, Japan

**Keywords:** neuroblastoma, screening, mortality, incidence, HPLC, rate ratio, Japan

## Abstract

**Background:**

Neuroblastoma (NB) is one of the most malignant neoplasms in childhood. In Japan, while a nationwide screening program at six months of age was introduced in 1985, its efficacy has not been systematically evaluated before or after its introduction. The screening test was changed from a qualitative method to a quantitative method (i.e., high performance liquid chromatography, HPLC) with higher test precision around 1990. However, the Japanese government stopped the program in 2003, after reports which did not show a reduction in mortality from NB. To evaluate the effectiveness of the program, a systematic large-scale epidemiological study was conducted.

**Methods:**

A retrospective cohort study was carried out to evaluate the effectiveness of the NB screening with HPLC test at 6 months of age in Japan, in comparing mortality and incidence of NB after 6 months of age between screened children and concurrent non-screened children in the same area. The study cohort was defined retrospectively as those children who were born after the introduction of HPLC test, from its earliest introduction of January 1984 to December 31, 1997, in twenty-five prefectures of Japan, which cover approximately half of the newborn population of Japan.

**Results:**

The study cohort consisted of 4.31 million. We identified 66 NB deaths in the study cohort for the analysis after 6 months. Kaplan-Meier estimate of cumulative mortality of NB per million children at 6 years was 15.33 for the screened group and 32.63 for the non-screened group, respectively. The difference of hazard between the two groups was statistically significant. The age specific mortality rate ratio of NB (95% confidence interval (CI)) was statistically lower at 1 - 3 years [0.415 (0.212 - 0.810)]. The rate ratio of NB incidence (95% CI) at the early stage (i.e., 1, 2 and 4S) between them was statistically higher at 6 months - 1 year [9.56 (4.76 - 19.23)]. That of NB incidence at the advanced stage (i.e., 3 and 4) was statistically lower at 1 - 4 years [0.40 (0.26 - 0.62)].

**Conclusion:**

The present study showed the reduction of mortality from NB, as well as the increase of the identification of early stage of NB and the decrease of advanced stage of NB. These findings strongly suggest the effectiveness of the NB screening with HPLC test in Japan. Although there could be several biases inherent to the study design, their possibilities are considered to be relatively low from observational information and theoretical consideration.

## 1. Introduction

Neuroblastoma (NB) is an embryonal tumor of the autonomic nervous system, and the most common extracranial solid tumor of children under the age of five. It affects one in 7,000 children and accounts for about 15% of cancer mortality in children (Young et al., 1987; Bernstein et al., 1992). The clinical presentation of NB is highly variable, ranging from a mass that causes no symptoms to a primary tumor that causes critical illness. Biologically favorable NB is diagnosed before one year of age, typically stays benign, and completely regresses spontaneously in many patients. However, about half of cases develop after the age of 18 months into metastatic disease, and have shown only modest improvement in survival, despite intensive treatment, over the past 25 years (Maris, 2010).

Most NBs produce catecholamines and their metabolites (i.e., vanillylmandelic acid and homovanillic acid) can be measured in urine samples to allow early detection of preclinical tumors in infancy (Sawada et al., 1982; Sawada et al., 1984). Then, screening programs for NB at 6 months of age were introduced in Japan in the early 1970s to aim at early diagnosis and treatment, and were extended nationwide in 1984. In 1997, 1.04 million infants (87% of all infants) in Japan participated in the screening program. This screening was also introduced in other countries (Schilling et al., 1994; Woods et al., 1996; Mathieu et al., 1996; Kerbl et al., 1998). In Japan, initially a qualitative test for vanillylmandelic acid in urine, such as a spot test and thin layer chromatography (TLC), was used. However, in 1990, this test was nationally replaced by quantitative measurements with high-performance liquid chromatography (HPLC), since the test accuracy of TLC was inferior to that of HPLC (Nishi et al., 1998; Sawada, 1988).

The screening program for NB inevitably over-diagnosed localized tumors with favorable prognoses, which included those which spontaneously regressed or matured without becoming clinically manifest (Maris, 2010; Murphy et al., 1991; Yamamoto et al., 1998). Therefore, the improvement of survival for NBs found by screening is not necessarily considered as effectiveness of screening programs, and the effectiveness of NB screening programs in Japan has been evaluated in terms of mortality reduction. While most of the studies were before and after or time series studies and only one retrospective cohort study was conducted, even as for the best study design, the results of these studies varied widely (Hisashige, 1999). Also, several methodological problems in evaluation for NB screening programs, including lack of adequate sample size, good documentation and well designed study, have been pointed out (Murphy et al., 1991; Treuner & Schilling, 1995; Report of the 1998 Consensus on Neuroblastoma Screening, 1999).

Two prospective large cohort studies for evaluation of NB screening in North America and Germany did not show a reduction of mortality from NB (Woods et al., 1996; Woods et al., 2002; Schilling et al., 2002). In response to them, in 2003, the Japanese government decided to halt screening, on the condition that rates of incidence and mortality should be assessed. However, the prospective cohort study in North America adopted TLC as a primary test for screening, for which accuracy was much lower than HPLC (Nishi et al., 1998, Nishi, 2013). Moreover, the effectiveness of TLC screening in terms of mortality has already been denied in Japan (Nishi et al., 1997). Therefore, the effectiveness of the recent screening program with HPLC in Japan has remained unanswered. As to the study in Germany, it is pointed out that there are serious problems in study design (i.e., control setting) and estimation of NB incidence (Nishi et al., 2003; Nishi, 2013). Therefore, its results and conclusions should be critically appraised.

Under these circumstances, it is necessary to evaluate the effectiveness of the screening program in Japan to determine whether it could decrease mortality of NB and/or incidence of advanced NB, even though neither a randomized controlled trial nor a prospective large cohort study is practically and politically feasible in Japan. Several attempts to evaluate the effectiveness of NB screening programs in Japan have been conducted before and after the decision to halt screening (Hisashige et al., 2000; Hiyama et al., 2008). We conducted a nationwide retrospective cohort study to evaluate the effectiveness of the NB screening program with HPLC test in Japan.

## 2. Methods

### 2.1 Study Design

A retrospective cohort study was carried out by collecting data of HPLC screening and NB patients in twenty-five prefectures of Japan, and comparing the mortality and incidence of NB after 6 months of age between screened children (participants of the program) and concurrent non-screened children (non-participants) in the same study population.

The study cohort was a dynamic population (Kleinbaum et al., 1982; Rothman et al., 1998) and defined retrospectively as those children who were born after the change of screening test from qualitative methods to HPLC in each area, from its earliest introduction of January 1984 to December 31, 1997. The study areas are geographically scattered from north to south, and consist of 25 prefectures from 8 districts: Hokkaido, Tohoku (Miyagi), Kanto (Tochigi, Gunma and Saitama), Chubu (Niigata, Ishikawa, Shizuoka, Aichi and Mie), Kinki (Shiga and Kyoto), Chugoku (Tottori, Hiroshima and Yamaguchi), Shikoku (Kagawa and Tokushima), Kyushu (Fukuoka, Saga, Nagasaki, Kumamoto, Oita, Miyazaki, Kagoshima and Okinawa). The population of these areas covered approximately half the newborn population of Japan (i.e., the 581,000 babies (49 percent) born in these areas of 1,192,000 born in Japan in 1997). A double screening program was started in Sapporo City, Hokkaido (at 6 and 14 months), Miyagi Prefecture (at 6 and 18 months), and Kyoto Prefecture (at 6 and 18 months) during the study period. The children born in those three areas after the introduction of the double screening program were excluded from the study cohort.

The sample size needed for the study was calculated in planning the study. The total number of person-years was 23 million when the rate ratio of NB mortality (screened children/non-screened children) was 0.6, and 8.4 million when the rate ratio was 0.4, assuming the mortality rate of NB in the non-screened children is 11 per million person-years, the proportion of those screened in the study population was 80 percent, the alpha of the statistical testing was one-sided 0.05, and the statistical power was 0.9.

### 2.2 Ascertaining Screening and Neuroblastoma

In defining screened and non-screened children, we based on the participant list of the screening program in each prefecture. Its existence and availability in the research areas were confirmed by the interim report of the Ministry of Health and Welfare. On the other hand, as to identification of NB patients, a study group member in each area surveyed NB patients by contacting with hospitals in the area, and confirming with NB patient data sources such as medical records of the National Medical Aid Program for Specific Chronic Pediatric Diseases, the Japan Children’s Cancer Registry, the prefecture Cancer Registry, and the prefecture Registry of Childhood Malignancies. Under these conditions concerning information availability, we selected 25 prefectures from all 47 prefectures.

The standardized patient report form was used, including gender, birthplace, information source, screened or non-screened at 6 months, results of the screening, date of diagnosis, occasion of diagnosis (screening or clinical symptoms), clinical stage at diagnosis (INSS), alive or dead on December 31, 1998, date of death, and causes of death. The regional investigators filled out the report form for each identified patient and sent it to the study coordination office (Department of Preventive Medicine, School of Medicine, Tokushima University).

The cause of death was classified by the regional investigators as 1) death from NB, 2) death related to treatment for NB, 3) death from other cancers, or 4) other causes. We defined NB death as death from NB or death related to treatment for NB.

Incidence cases of NB were classified according to the Evans staging system or International Neuroblastoma Staging System (INSS; panel) (Evans et al., 1971; Brodeur et al., 1993). While NB at the stages 1, 2 and 4S are localized and treated by surgery or surgery with chemotherapy, NB at the stages 3 and 4 are non-localized or disseminated and treated according to the protocol by the Japanese Neuroblastoma Study Group (Sawaguchi et al., 1990). In the case of stage 3, patients younger than 12 months were treated in the same way as the former. According to Evans (Evans et al., 1971), in this study, NB was classified as an early stage for the stages 1, 2 and 4S, and as an advanced stage for the stages 3 and 4. In addition, NB at stage 4 was independently analyzed as the most advanced stage with poor prognosis.

### 2.3 Statistical Analyses

Firstly, the cumulative mortality of NB for the screened and non-screened groups was analyzed by the Kaplan-Meier method, and the two hazards were compared by Generalized Wilcoxon test and Log-Rank test. An age-specific NB mortality rate for the two groups and the mortality rate ratio (screened / non-screened), as well as their corresponding 95% confidence intervals, were calculated based on the person-years of observation from six months old (Klenbaum et al., 1982). NB patients diagnosed clinically by the age of 6 months were excluded from the analysis.

Secondly, an age-specific NB incidence for the screened group and non-screened group and the incidence rate ratio (screened/non-screened), as well as their corresponding 95% confidence intervals, were calculated based on the person-years of observation from six months old (Klenbaum et al., 1982).

## 3. Results

### 3.1 Study Population

The study cohort consisted of 4.31 million children born in the twenty-five prefectures, 3.71 million children (86%) in the screened group and 0.60 million children (14%) in the non-screened group (Table 1). The total person-years observed was 22.88 million (19.53 million person-years for the screened group and 3.34 million person-years for the non-screened group). The mean time of observation from 6 months was 5.3 years. In total, 957 NB patients in the study population were reported by the regional investigators. Eighty-seven cases died by the end of 1998.

**Table 1:** Study cohort and person-years of observations after HPLC introduction

Item	Screened	Non-screened	Total
Number of children	3,705,670	603,900	4,309,570
Number of person-years	19,532,468	3,343,332	22,875,800

### 3.2 Deaths and Incidence Cases of Neuroblastoma

In total, 87 children died by the end of 1998. Of the 87 deaths, 14 cases were detected clinically before 6 months (5 cases died before 6 months and 8 cases were alive at 6 months) and 7 cases died of causes other than NB. In addition, detailed information for one case could not be confirmed, and it was excluded from the analysis. Therefore, 66 NB deaths were identified in the study cohort after 6 months. Forty-three cases were boys, and 23 were girls.

As is shown in Table 2, forty-nine cases were in the screened group, and 17 cases were in the non-screened group. Participation in the screening program was confirmed from the participants list of the program for 42 cases (64%), by interviewing parents for 21 cases (32%), and by other medical records for 3 cases (5%). The number of deaths from NB at the age from 6 months to 1 year was 2 in the screened group and 1 in the non-screened group. The deaths were frequently identified at the age from 1 to 3 years, 31 cases in the screened group and 12 cases in the non-screened group. After the age of 4 years, 16 cases in the screened group and 4 cases in the non-screened group were observed (Table 2).

**Table 2:** Deaths and incidence cases of neuroblastoma classified by age

	Screened children	Non-screened children	Total

	(N=3,705,670)	(N=603,900)	(N=4,309,570)
**Age at death**			
6 months - 1 year	2	1	3
1 - 3 years	31	12	43
1 year old	*6*	*5*	*11*
2 years old	*11*	*3*	*14*
3 years old	*14*	*4*	*18*
4 - 7 years	15	3	18
8 years -	1	1	2
Total	49	17	66
**Age at incidence**			
6 months - 1 year	602	20	622
Early stage (1, 2, 4s)	*472*	*8*	*480*
Advanced stage (3, 4)	*130*	*12*	*142*
1 - 4 years	98	36	134
Early stage (1, 2, 4s)	*31*	*8*	*39*
Advanced stage (3, 4)	*67*	*28*	*95*
5 - 7 years	7	3	10
Early stage (1, 2, 4s)	*0*	*0*	*0*
Advanced stage (3, 4)	*7*	*3*	*10*
8 years -	1	0	1
Early stage (1, 2, 4s)	*0*	*0*	*0*
Advanced stage (3, 4)	*1*	*0*	*1*
Total	708	59	767

In total, 767 incidence cases were identified in the study cohort after 6 months, excluding 190 cases which did not correspond to the study area and birth cohort defined. Table 2 shows the incidence cases of NB classified by age and stage. Seven hundred and eight cases were in the screened group and 59 cases were in the non-screened group. As to information source for NB, hospitals, which dealt with them, were 72% (550 cases) and registration data bases were 28% (217 cases). Participation in the screening program was confirmed from the participants list of the program for 565 cases (74%), by interviewing parents for 184 cases (24%), and by other medical records for 18 cases (2%).

The number of incidence cases of NB from 6 months to 1 year was 602 in the screened group and 20 in the non-screened group. The number of the cases dramatically decreased in both groups: at the age from 1 to 4 years, 98 cases in the screened group and 36 cases in the non-screened group; after the age of 5 years, 8 cases in the screened group and 3 cases in the non-screened group. While the number of NB in both early and advanced stages showed the same trend among the screened group, that in the advanced stage among the non-screened group increased at the age from 1 to 4 years old (Table 2).

### 3.3 Mortality of Neuroblastoma

**Figure 1:**
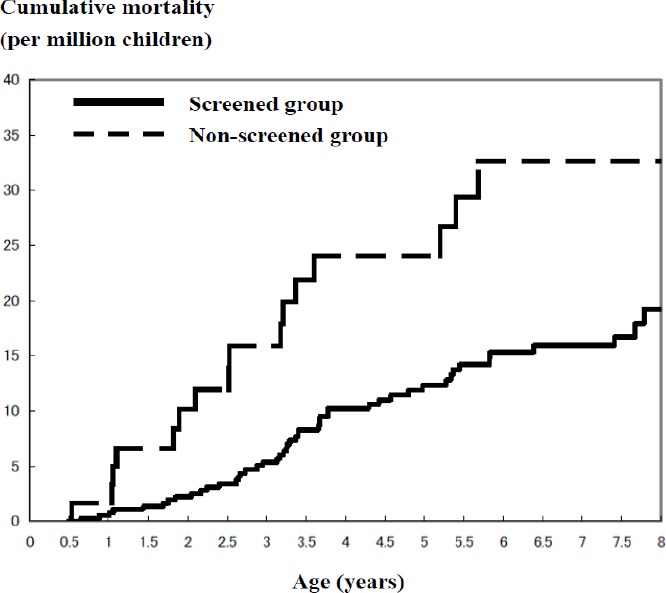
Cumulative mortality from neuroblastoma in screened and non-screened group (Kaplan-Meier estimates)

Cumulative mortality curves after 6 months in the screened group and the non-screened group are illustrated in Figure 1. The difference in cumulative mortality between the two curves increases to around the age of 4 years. The difference was continuously maintained after the age of 4 years. The difference of hazard between the two groups was statistically significant (Generalized Wilcoxon test: χ^2^=8.278, p=0.0040; Log-Rank test: χ^2^=7.134, p=0.0076). In the screened group, cumulative mortality rate per million children increased from 2.22 at 2 years to 19.17 at 8 years. In the non-screened group, the rate increased form 10.16 at 2 years to 32.63 at 8 years.

As to age specific mortality rate from NB (Table 3), in each group, mortality rate of NB was highest at the age of 1 - 3 year. The mortality rate was lower in the screened group and its rate ratio was less than 1 in all age categories. A statistically significant reduction of the rate ratio was observed at the age of 1 - 3 years [0.415 (0.212 - 0.810)].

**Table 3:** Neuroblastoma mortality rate per million person-years and its rate ratio

Age	Screened children	Non-screened children	Rate ratio
6 months - 1 year	1.08 (0.00 - 2.58)	3.31 (0.00 - 9.80)	0.326 (0.030 - 3.595)
1 - 3 years	3.11 (2.00 - 4.23)	7.51 (3.26 - 11.76)	0.415 (0.212 - 0.810)
4 - 7 years	2.17 (1.07 - 3.27)	2.46 (0.00 - 5.25)	0.880 (0.255 -3.040)
6 months - 7 years	2.46 (1.76 - 3.15)	4.50 (2.22 - 6.77)	0.547 (0.306 - 0.976)

(): 95% confidence interval.

### 3.4 Incidence of Neuroblastom

Table 4 shows the incidence rate of NB and its rate ratio (95% CI), classified by age between the screened group and the non-screened group. At the age of 6 months - 1 year, the incidence rate of NB per million person-years was statistically higher among the screened group (325.9), in comparison with the non-screened group (66.8). The rate ratio (95% CI) of incidence rate was statistically higher [4.88 (3.13 - 7.62)]. In contrast, at the age more than 1 year, the rate was less than 1. Statistically significant decrease was observed at 1 - 4 years [0.45 (0.31 - 0.67)].

**Table 4:** Incidence of neuroblastoma and its rate ratio classified by age

Age	Number of person-years (million)	Incidence rate (per million person-years)		Rate ratio (95% CI)

		Screened children	Non-screened children	
6 m - 1 year	2.15	325.92	66.80	4.88 (3.13 - 7.62)
1 - 4 years	14.04	8.14	17.93	0.45 (0.31 - 0.67)
5 - 7 years	5.31	1.55	3.74	0.48 (0.11 - 1.61)

Rate ratio: screened/non-screened, CI: confidence interval.

As is shown in Table 5, the rate ratio of NB incidence (95% CI) at the early stage (i.e., 1, 2 and 4s) between the screened and the non-screened groups was statistically higher at the age of 6 months - 1 year [9.56 (4.76 - 19.23)]. The rate at the advanced stage (i.e., 3 and 4) were statistically lower at 1 - 4 years [0.40 (0.26 - 0.62)]. In the case of NB at the stage of 4 only, it was less than 1 in all age categories. Statistically significant decrease was observed at the age of 1 - 4 years [0.34 (0.20 - 0.58)].

**Table 5:** Incidence of neuroblastoma and its rate ratio classified by stage and age

Age	Incidence rate (per million person-years)		Rate ratio (95% CI)

	Screened children	Non-screened children	
**Early stage (1, 2, 4s)**			
6 m - 1 year	255.54	26.72	9.56 (4.76 - 19.23)
1 - 4 years	2.58	3.98	0.65 (0.30 - 1.41)
5 - 7 years	0	0	NA
**Advanced stage (3, 4)**			
6 m - 1 year	70.38	40.08	1.76 (0.97 - 3.17)
1 - 4 years	5.57	13.94	0.40 (0.26 - 0.62)
5 - 7 years	1.55	3.74	0.42 (0.11 - 1.61)
**Stage 4**			
6 m - 1 year	19.49	23.38	0.83 (0.37 - 1.87)
1 - 4 years	3.57	10.46	0.34 (0.20 - 0.58)
5 - 7 years	1.55	2.49	0.62 (0.13 - 3.00)

Rate ratio: screened/non-screened, CI: confidence interval.

## 4. Discussion

In this study, the screening program for NB with HPLC test in Japan showed a significant reduction in mortality of NB. Also, the screening identified a large number of NB in the early stage. Then it decreased the incidence of NB in the advanced stage. Since this study was a nationwide epidemiological study based on a large-scale population, these findings strongly suggest the effectiveness of the NB screening program, realizing early detection and treatment. However, there could be several biases such as selection and/or information biases, which are inherent to the study design. Therefore, the results need to be critically evaluated.

As to study design, ideally, a randomized controlled trial (RCT) would be desirable rather than a cohort study to prevent selection bias and confirm effectiveness of a screening. However, since the incidence and mortality of NB are considerably low, it would not be feasible or appropriate to do a usual RCT. In addition, in Japan, without comprehensive evaluation, health technologies have been introduced, particularly in screening areas including NB screening (Hisashige, 1994; 2009). Under such circumstances, the second best study design is a cohort study. In Japan, the possibility or chance for a relatively high-quality study is afforded by conducting a retrospective cohort study, comparing the screened with concurrent non-screened children in the same study population.

Table 6 summarizes the results of cohort studies internationally available. In critically appraising them, we also examined the issues around our study. In other countries, two large prospective cohort studies were conducted to evaluate the effectiveness of NB screening, before its introduction. The North American study, a prospective population-based cohort study, reported no positive effects on the incidence of unfavorable advanced-stage NB and no reduction in mortality (Woods et al., 1996; 2002). However, the screening test adopted as a primary test was TLC test, of which sensitivity and specificity were much lower than the present screening test, HPLC (Nishi et al., 1998; Nishi, 2013). Moreover, the effectiveness of TLC screening has already been denied even by a before-after study (Nishi et al., 1997). In addition, this study adopted gas-chromatography/mass-spectrometry as a secondary test. This kind of sequential testing only decreases sensitivity, even though it increases specificity (Sackett et al., 1991). In addition, since the comparison was done between screened and control regions rather than screened and non-screened individuals in each area, confounding and bias would be inevitable. Also, the sample size seems to be not large enough for evaluating effectiveness.

**Table 6:** Cohort studies evaluating neurobalstoma screening

Article	Screening program	Study design	Main results	Issues addressed
***Prospective cohort study***				
Woods 2002	Screening test: TLC (primary) GCMS (secondary) Screening age: 3 weeks and 6 months Area: Quebec, Canada	Design: prospective cohort study Subject: 476,654 (all children in Quebec) Control: Ontario, Canada, Canada excluding Quebec; Minnesota and Florida, Greater Delaware Valley, US Outcome information: tumor registries	Standardized mortality ratio: NS (0.90 - 1.39) Standardized incidence ratio: NS (0.81 - 1.40)	Low accuracy of TLC test Without concurrent control Small sample size Insufficient coverage of tumor registry
Schilling 2002	Screening test: HPLC Screening age: one year Area: Germany	Design: prospective cohort study Subject: 2,581,188 (Mainly old West Germany) Control: 2,117,600 (Mainly old East Germany) Outcome information: tumor registries	Mortality of NB: similar between two groups, NS (relative risk = 1.1) Incidence of stage 4 NB: similar between two groups NS (relative risk =1.0)	Less comparability of subject areas Without concurrent control Invalid estimates Insufficient coverage of tumor registry
***Retrospective cohort study***				
Hiyama 2008	Screening test: HPLC or qualitative Screening age: 6 months Area: Japan	Design: retrospective cohort study with before-after comparison Subject: 10,868,86 (HPLC), and 5,299,412 (qualitative) Control: 6,130,423 (before screening), Outcome information: tumor registries	Mortality of NB: lower in HPLC group (p<0.0001, relative risk =0.53), qualitative group (p<0.005, relative risk =0.73) Incidence of stage 4 NB: lower in HPLC group (p=0.003, relative risk =0.75)	Less comparability of subject in different time Without focusing concurrent control Insufficient coverage of tumor registry
Yamamoto 2002	Screening test: HPLC or qualitative Screening age: 6 months Area: 7 prefectures, Japan	Design: retrospective cohort study with before-after comparison Subject: 550,331 (HPLC), and 1,142,519 (qualitative) Control: 713,025 Outcome information: tumor registries	Mortality of NB: lower in HPLC group (NS, relative risk =0.62), qualitative group (NS, relative risk =0.66) Incidence of NB: higher in HPLC group (p<0.0001, relative risk =2.6), qualitative group (p=0.01, relative risk =1.4)	Small sample size Without focusing concurrent control Insufficient coverage of tumor registry
Suita 1996	Screening test: HPLC Screening age: 6 months Area: Kyushu district, Japan	Design: retrospective cohort study Subject: 484,599 Control: 92,966 Outcome information: tumor registries	Mortality of NB: NS (relative risk =0/1.4) Incidence of NB: higher (p<0.01, relative risk =6.4 at the age of 6 months - 1 year, NS, relative risk =1.3 at the age of 1 - 5 years)	Small sample size Insufficient coverage of tumor registry
Nishi 1993	Screening test: HPLC or qualitative Screening age: 6 months Area: Sapporo city, Japan	Design: retrospective cohort study Subject: 73,226 Control: 24,636 Outcome information: tumor registries	Mortality of NB: NS (relative risk =0.56) Incidence of advanced NB: NS (relative risk =0.84)	Small sample size Insufficient coverage of tumor registry

NB: neuroblastoma, NS: not significant, TLC: thin-layer chromatography, GCMS: gas-chromatography/mass-spectrometry, HPLC: high-performance liquid chromatography.

On the other hand, a prospective cohort study in Germany for evaluating a screening program with HPLC test at 12 months of age indicated no reduction of mortality (Schilling et al., 2002). However, it is pointed that there are serious problems in the study design and the analysis of results (Nishi et al., 2003). For example, the randomization of study areas was not done. While control areas were located mainly in old East Germany, the subject areas were located in old West Germany. This selection of study areas could introduce biases for identification and management of NB. In this study, incidence of NB in the control was relatively low compared with that generally reported (Nishi et al., 2003; Hiyama et al., 2008). Therefore, the results of the study still remain inconclusive.

In comparison with a prospective cohort study, a retrospective cohort study is latently more liable for biases. Recently, a large before-after study based on a retrospective cohort was reported in Japan (Hiyama et al., 2008). This study showed that the mortality rate from NB in the cohort at the screening period was lower than that in the cohort at the prescreening period, especially in the period by quantitative HPLC. The relative risk was 0.53 for the HPLC period. This estimate was similar to that of our study, although there was no direct comparison between screened and non-screened groups. However, there must be some bias due to the changes in the background of healthcare around NB during the 20-year research period. Also, the reliability and validity of available information in diverse subject areas were not confirmed.

There were several small retrospective cohort studies (Nishi et al., 1993; Suita et al., 1998; Yamamoto et al., 2002). These studies did not show a significant reduction in mortality of NB. Their most serious problem is that the sample size was too small to detect a difference. As mentioned before, tens of million person-years were needed by the sample size estimation prior a study. In our study, based on this estimate, a sufficient sample was assured by collecting data systematically and collaboratively from 25 prefectures in Japan.

Besides these cohort studies, several time-series and/or before-after studies, which are indirect evaluation and highly susceptible to a lot of biases, were conducted. The analysis of mortality from NB based on national vital statistics indicated a significant reduction of mortality at the age of 1 - 4 years after the introduction of NB screening in Japan, which clearly showed a contrast to that in France without NB screening (Nishi et al., 2004; Nishi, 2013). Based on a cancer registry, a before-after study of mortality and incidence of NB in Osaka, Japan (Ajiki et al., 1998) showed a significant but small reduction of mortality after the introduction of NB screening. However, a comparison of mortality between Osaka and the UK (Honjyo et al., 2003) showed no difference in mortality and incidence of NB between these areas. The reason for difference between national vital statistics and a regional registry study is that migration (moving in and/or out) of residents is frequent and large in the metropolitan areas such as Tokyo and Osaka. Therefore, cancer registration in one area is not reliable or valid enough to capture all cancers among residents. Then, it is pointed out that mortality rate and/or incidence rate were relatively low in Osaka, compared with those in past studies in Japan (Nishi et al., 2000). This is the reason why Osaka and Tokyo were excluded from our study areas.

As to this selection (migration) bias, which will emerge from migration to other area among subjects, in our study, NB incidence and death were identified by collecting information from hospitals and cancer registry databases among half the population of Japanese children. It would minimize the bias. Althogh this approach, made it possible to obtain accurate and sufficient clinical information, it did not necessarily guarantee the ascertainment of complete NB deaths in the study population. Therefore, there might be a bias (Kleinbaum et al., 1982). An alternative for confirmation of NB deaths is the death certificate for the the national vital statistics. In comparison with a prospective cohort study, using death certificate (Hisashige et al., 2001), mortality rate in our study is little lower than or almost identical to the former. Even if there were missing or migrating cases, it would not be causally related to screening participation. Therefore, it would be considered as non-differential misclassification (Kleinbaum et al., 1982; Rothman et al., 1998).

Besides biases examined above, several other biases should be considered. Main biases in screening evaluation are pointed out such as lead-time, length time, overdiagnosis, and susceptibility biases (Sackett et al., 1991). However, the former three biases, which are inevitable for survival analysis, were eliminated in this study, since mortality was used as an index of effectiveness for screening. As to susceptibility (selection) bias, it is also unlikely to be identified, because risk factors for NB have not been established yet (Ferrís i Tortajada et al., 2005; Heck et al., 2009). Moreover, major difference between screened and non-screened groups in Japan was reported only in maternal age, number of parity, full-time occupation, and family structure (Makimoto et al., 2001).

On the other hand, as to information bias, the ascertainment of participation in screening should be examined. In the study, participation in the screening program was confirmed from the participants list for 74%, and by interviewing parents for 24%. In general, interviews are susceptible to memory or recall bias. However, in Japan, participation in NB screenings was recorded in a maternal child health handbook, which is distributed to all pregnant women and consists of records of pregnancy, delivery and child development and healthcare. This situation is quite different from general cancer screenings. Therefore, the memory bias would be unlikely to occur in NB screening.

The findings and discussions mentioned above in the present study strongly suggest the effectiveness of the NB screening with HPLC test at the age of 6 months in terms of mortality and incidence of NB. In addition, the nationwide prospective cohort study for this screening has been conducted and its interim report showed also significant reduction of NB mortality (Hisashige et al., 2001). On the other hand, the mortality of NB in Japan has been increasing after the discontinuance of NB screening (Nishi, 2013). In taking the evidence of our studies in Japan, as well as other various domestic and international evidence critically appraised, into consideration, it is necessary to discuss whether NB screening should be re-examined and re-started on nationwide scale in Japan.

In this examination, an additional issue to be addressed is the overdiagnosis of clinically favorable NB, which is inevitable for NB screening. It is impossible to distinguish favorable NB overdiagnosed from progressive NB among NB detected by screening. Even though harm due to treatment for this type of NB was included in terms of mortality in our study, quality of life and psychophysical disability were not considered. Therefore, in taking spontaneous regression among favorable NB into consideration, both the treatment strategy and the appropriate screening period should be examined. For example, the adoption of wait-and-see strategies suggested the prevention of unnecessary treatment for selected cases (Oue et al., 2005). Also, the screening program changing its period from 6 months to 14 or 18 months showed that the program at 18 months detected more cases with unfavorable prognosis than those at 6 and 14 months (Hanai et al., 2010).

In addition, for nationwide screening programs, their economic efficiency must be considered as well as their effectiveness under the financial pressure. Cost-benefit analysis of NB screening, which was based on the past evidence for effectiveness, indicated that costs and benefits per NB case finding by HPLC screening were $138,800 and $170,800, respectively (Hisashige et al., 1994). Net benefit was $3,200. Although there would be no major change in conclusion, further studies would provide more accurate estimates based on recent studies in Japan.

## 4. Conclusion

The present study showed the reduction of mortality from NB, as well as the increase of the identification of early stage of NB and the decrease of advanced stage of NB. These findings strongly suggest the effectiveness of the NB screening with HPLC test at age of 6 months in Japan, realizing early detection and treatment. There could be several biases such as selection and/or information biases, which are inherent to the study design. However, the possibilities of these biases are considered to be relatively low from observational information and theoretical consideration.

## Appendix: Neuroblastoma Screening (NBS) Evaluation Group

**Principal researcher**

Hisashige, A. (Department of Preventive Medicine, Tokushima University)

**Collaborative researchers**

Mikasa, H. (Department of Preventive Medicine, Tokushima University), Hayashi, K. (School of Health Sciences, Gunma University)

**Research members and/or regional investigators**

Asami, N. (Department of Pediatrics, Niigata University), Hayashi, Y. (Department of Pediatric Surgery, Tohoku University), Hiyama, E. (Department of Pediatrics, Hiroshima University), Horibe, K. (Department of Pediatrics, Nagoya University), Hyakuna, N. (Department of Pediatrics, University of the Ryukyus), Katanoda, K. (School of Medical Sciences, University of Tokyo), Kawakami, T. (Department of Pediatrics, Tottori University), Ohta, S. (Department of Pediatrics, Shiga University of Medical Science), Nishi, M. (Department of Public Health, Sapporo Medical University), Sawada, T. (Department of Pediatrics, Kyoto Prefectural University of Medicine), Sugita, K. (Department of Pediatrics, Dokkyo University), Suita, S. (Department of Pediatric Surgery, Kyushu University), Takeda, T. (Sapporo South National Hospital), Yamamoto, K. (Saitama Children’s Medical Center).

(Each affiliation among the research group members was at the time of the research.)
